# Editorial: Green and sustainable extraction techniques for bioactives in food, plants, pharmaceuticals, and cosmetics

**DOI:** 10.3389/fchem.2024.1388362

**Published:** 2024-02-28

**Authors:** Prince Chawla, Rahmatuzzaman Rana, Kandi Sridhar, Baskaran Stephen Inbaraj

**Affiliations:** ^1^ Department of Food Technology and Nutrition, Lovely Professional University, Phagwara, Punjab, India; ^2^ School of Dentistry and Medical Sciences, Charles Sturt University, Wagga Wagga, NSW, Australia; ^3^ Department of Food Technology, Karpagam Academy of Higher Education (Deemed to be University), Coimbatore, India; ^4^ Department of Food Science, Fu Jen Catholic University, New Taipei City, Taiwan

**Keywords:** green extraction, eco-friendly solvents, bioactive compounds, agri-pharma-food applications, green solvents

More recently, there has been a significant surge in green and sustainable analytical approaches for the analysis of bioactive phytochemicals in various sectors including food, plants, pharmaceuticals, and cosmetics. The concept of “Green Analytical Chemistry” was initially introduced by de la Guardia and Ruzicka in 1995 with the primary objective of downsizing analytical procedures while minimizing the use of reagents and chemicals. Moreover, key principles such as miniaturization, automation, utilization of sustainable solvents, and reduction in energy consumption are fundamental for achieving green analytical chemistry objectives. To ensure the overall greenness of the analytical process, it is imperative to consider the reduction and/or replacement of existing extraction techniques with environmentally sustainable alternatives. This transition is pivotal in mitigating environmental impact. A critical component in attaining this objective involves choosing an appropriate solvent for extraction processes, as the type of solvent utilized can significantly impact the effectiveness of isolating bioactive/nutrient components, energy consumption, and emissions. Therefore, the development of eco-friendly and sustainable extraction methodologies is currently a prominent research focus within the multidisciplinary field of applied chemistry, such as food science, pharmaceuticals, cosmetics, and beyond. Hence, three primary strategies have been identified for designing and implementing green extraction methods on both laboratory and industrial scales, with the aim of optimizing the utilization of raw materials, solvents, and energy: 1) improving and optimization of existing processes; 2) using non-dedicated equipment; and 3) innovation in processes and procedures but also in discovering alternative solvents. To promote these strategies, a consortium of interdisciplinary experts (Dr. Kandi Sridhar, Dr. Baskaran Stephen Inbaraj, Dr. Prince Chawla, and Er. Rahmatuzzaman Rana), specializing in green extraction technologies, has organized a specialized Research Topic focused on “*Green and Sustainable Extraction Techniques for Bioactives in Food, Plants, Pharmaceuticals, and Cosmetics*” to be published in Frontiers in Chemistry (ISSN 2296–2646), an international open access journal that explores the role of chemistry in our everyday lives. In this Special Research Topic, we explored the forefront of research and innovations in green chemistry, uncovering their vast potential through the curation of research and review articles encompassing, but not limited to:

• Green and sustainable analytical chemistry and eco-friendly solvents
• Green solvents in food analysis and extraction of phytochemicals
• Green extraction methods/techniques for bioactives/micronutrients from food/agro byproducts, fruits and vegetables, plants/plant products, cosmeceuticals, pharmaceuticals, and food/agro byproducts and wastes

For this Special Research Topic, esteemed researchers worldwide were invited to contribute, culminating in a total of 9 submissions dealing with green extraction technologies for bioactive compounds and their applications in agri-food-pharma, and cosmetic industries. Following a rigorous single-blind peer-review process, 6 articles were deemed suitable for final publication. One of the studies under this Research Topic, investigated by Singh et al., reviewed the extraction of high-value bioactive components from grape leaves using conventional (Soxhlet extraction, classic solvent extraction, reflux extraction, and maceration) and non-conventional (microwave-assisted extraction, ultrasound-assisted extraction, and supercritical fluid extraction) extraction techniques. Additionally, the review emphasized the development of advanced green technologies for extraction, aiming to achieve clean label status and safety with increased yield and efficiency, as well as minimize impact on product quality. Another study by Xu et al. developed an efficient approach utilizing vortex-assisted matrix solid phase dispersion coupled with ultra-high-performance liquid chromatography-triple quadrupole mass spectrometry (VA-MSPD-UHPLC-MS/MS) for the simultaneous extraction and determination of 7 alkaloids and 3 organic acids from *Uncariae Ramulas Cum Unicis*. The optimal extraction conditions were determined through Box-Behnken design combined with response surface methodology. The recoveries of the 10 bioactive compounds from *Uncariae Ramulas Cum Unicis* ranged from 95.9% to 103%, with intra-day and inter-day precisions exhibiting relative standard deviation of <2.97%. Overall, this method not only reduced solvent and sample consumption but also minimized sample processing and analysis time. Thus, the developed VA-MSPD-UHPLC-MS/MS method offers a successful and efficient means for extracting and analyzing the bioactive components from *Uncariae Ramulas Cum Unicis*.

Likewise, Tipare et al. used green hydrodistillation technology to extract bioactive compounds from *Milletia pinnata* oil and *Nardostachys jatamansi* for the development of pectin-crosslinked carboxymethyl cellulose/guar-gum nano hydrogel. The study showed the presence of spirojatamol and hexadecanoic acid methyl ester in both oil and extract. The hydrogel exhibited a high thermal stability and demonstrated bactericidal and anti-inflammatory activities against both Gram-positive and Gram-negative bacteria. The study concluded that the green hydrodistillation technology can facilitate the extraction of bioactive compounds, enabling the formulation of biocompatible and hydrophobic nanohydrogels capable of absorbing target-specific drugs, thereby offering promising solutions for addressing infections caused by pathogenic microorganisms. Similarly, Rahmani et al. investigated the anticancer and antibacterial effects of hydroethanolic extracts of Nettle (*Urtica dioica*) and Wormwood (*Artemisia absinthium*), as well as their nanoemulsion and nanoencapsulation forms. The study revealed that both nanoemulsion and nanoencapsulation forms with the particle size respectively ranging from 10 to 50 nm and 60–110 nm can effectively inhibit the growth of bacteria with the minimum inhibitory concentration (MIC) ranging from 11.25 to 95.00 μg/mL and minimum bactericidal concentration (MBC) ranging from 11.25 to 190.00 μg/mL. Additionally, exposure to their high concentration significantly lowered the viability of colon cancer cells HCT116 compared to other groups.

In another study, authors focused on isolation of specific bioactive compound, puromycin, from a novel *Streptomyces albofaciens* strain MS38 using fermented broth through extraction with n-butanol (Singh et al.). The puromycin exhibited a significant antimicrobial efficacy against various pathogenic bacteria and fungi due to presence of distinctive functional groups with potent antimicrobial attributes. Another study by Ahmad et al. extracted the uranium (VI) from aquatic solution of *Albizia Lebbeck* pods using biowaste-derived active carbon. The optimal extraction efficiency of 90.60% was achieved at a dose of 0.5 g of biowaste active carbon, pH of 6, contact time of 120 min, and uranium (VI) concentration of 10 ppm. The biowaste active carbon was also shown to possess both regeneration and reusable characteristics for uranium (VI) extraction across multiple sorption-desorption cycles, retaining its extraction capacity without significant loss. The development of efficient adsorbent from biowaste represents a promising advancement for addressing the substantial demand for uranium (VI) in nuclear energy applications.

In conclusion, the aforementioned studies collectively offer diverse strategies for the green extraction methodologies in promoting sustainability and innovation within the chemical industry. We are firmly convinced that these articles constitute a valuable repository of information on approaches to promote environmentally-friendly practices and enhance product quality. The vision underpinning this special Research Topic also paves the way for further exploration of sustainable way of extracting bioactive compounds from diverse sources. We look forward to continuing this important discussion in future special Research Topics.

